# Effects of *ompR* Deletion on Stress Tolerance and Virulence in *Salmonella* Typhimurium Monophasic Variant

**DOI:** 10.3390/microorganisms14071503

**Published:** 2026-07-09

**Authors:** Chunyu Xia, Zhuosi Li, Qingli Dong, Meirong Luo, Tingyu Liu, Binru Gao, Jiayi Xue, Yue Ma, Xuejuan Xia, Xiaojie Qin

**Affiliations:** School of Health Science and Engineering, University of Shanghai for Science and Technology, Shanghai 200093, China; 254312352@st.usst.edu.cn (C.X.); lizhuosi@usst.edu.cn (Z.L.); qdong@usst.edu.cn (Q.D.); 18084293598@163.com (M.L.); liuutingyu@163.com (T.L.); 251310259@st.usst.edu.cn (B.G.); 243452602@st.usst.edu.cn (J.X.); yuema@usst.edu.cn (Y.M.); xiaxuej1989@163.com (X.X.)

**Keywords:** *Salmonella* Typhimurium monophasic variant, outer membrane protein OmpR, food/food-processing relevant stresses, tolerance, virulence

## Abstract

*Salmonella* Typhimurium monophasic variant (STm) has emerged as a novel predominant serovar in global foodborne *Salmonella* outbreaks. In our previous study, the two-component regulator OmpR was significantly upregulated in STm under disinfectant stress. However, effects of *ompR* deletion on stress tolerance and pathogenicity of this pathogen remained unclear. Therefore, this study aimed to systematically investigate the function of *ompR* in the adaptation of STm to food/food processing environments (e.g., disinfectants, thermal treatment, and high-salt preservation) and virulence. An isogenic Δ*ompR* mutant was constructed using homologous recombination, and its phenotypic changes relative to the wild-type strain were evaluated, including bacterial growth, motility, stress resistance (thermal, osmotic, acid, and oxidative stress), and in vivo virulence in ICR mice. The results revealed that the deletion of *ompR* significantly decreased bacterial tolerance to multiple tested stresses, including thermal (50 °C, 55 °C, 60 °C), osmotic (10%, 20%, 30% NaCl), acid (pH = 3, 4, 5), and oxidative (1 mM, 2 mM, 5 mM H_2_O_2_) stresses. Additionally, the Δ*ompR* mutant exhibited markedly reduced swimming and swarming motility compared to the wild-type strain. In the ICR mice infection model, the Δ*ompR* mutant showed significantly attenuated virulence (100% survival rate within 7 days post-infection), with significantly lower bacterial loads in the liver, spleen and intestine. Transcriptome analysis showed that compared with the wild-type strain, the Δ*ompR* mutant strain exhibited significant upregulation of fimbriae-related genes and downregulation of flagellar biosynthesis and virulence-related genes, thereby affecting bacterial adhesion, biofilm formation, flagellar assembly and virulence expression. Collectively, these findings suggest that *ompR* contributes to the adaptation of STm to food/food processing environments and virulence, and this gene may provide potential targets for future research to control STm contamination in food production systems.

## 1. Introduction

*Salmonella* Typhimurium monophasic variant (STm), also designated as *Salmonella enterica* serovar 1,4,[5],12:i:-, has evolved from *Salmonella enterica* serovar Typhimurium through complex genetic mechanisms, such as the insertion of multidrug-resistant cassettes that replace the genomic region encoding phase II flagellar antigens [1,2,3]. This genetic modification not only abolishes phase II flagellar antigen expression but also confers enhanced multidrug resistance [4], rendering STm a major threat to public health. Over the past two decades, STm has undergone rapid global dissemination across Europe [5,6], Africa [7], and Asia [8], emerging as one of the predominant serovars responsible for human and animal salmonellosis [9,10].

Notably, STm is primarily transmitted through the food chain, with pork and pork products identified as its major reservoirs [11,12,13]. Recent high-profile outbreaks linked to STm-contaminated raw milk and chocolate have been reported worldwide [14,15]. In China, swine and swine-derived products accounted for 87% (222/255) of STm contamination cases between 2010 and 2018 [16], and STm has become the most prevalent *Salmonella* serovar isolated from diarrheal patients in several regions [17,18]. The persistent prevalence of STm in both food production environments and clinical settings highlights an urgent need to address its transmission and pathogenicity, particularly given its unique genetic traits that exacerbate control challenges. A key factor contributing to the successful persistence of STm in the food chain and subsequently pathogenicity is its remarkable tolerance to food- and food-processing-relevant stresses [19], including thermal treatment (e.g., pasteurization), high-salt preservation, acidic food matrices, and disinfectants [20]. This stress resilience enables STm to survive in harsh food chain environments and the gastrointestinal tract.

Our previous study demonstrated that repeated exposure of STm to benzalkonium chloride (BC) can induce stable adaptive resistance [21], further complicating its control in food production facilities. However, the molecular mechanisms underlying the stress tolerance of STm and the synergistic regulation with virulence remain poorly understood, limiting the development of effective control strategies.

Outer membrane proteins (OMPs) are critical for Gram-negative bacteria like *Salmonella*, serving as the primary interface between the bacterium and its environment [22,23]. They regulate outer membrane integrity, selective permeability, stress adaptation, and host/pathogen interactions, thereby influencing both environmental survival and virulence [24]. Among OMP-related regulators, OmpR-a response regulator of the EnvZ/OmpR two-component system (TCS), is highly conserved in pathogenic bacteria, including *Escherichia coli* [25], *Pseudomonas* spp. [26], and various *Salmonella* serovars [27]. In traditional *Salmonella* serovars, OmpR has been shown to regulate outer membrane pore protein expression and modulate physiological processes, including stress resistance and virulence [27]. However, despite its well-characterized roles in other *Salmonella* serovars, the specific function of OmpR in STm remains elusive.

Our previous transcriptomic analysis revealed significant upregulation of *ompR* in STm under BC stress, implying a potential role in disinfectant resistance. Therefore, this study aimed to systematically investigate the regulatory role of *ompR* in the environmental tolerance and virulence of STm. We constructed an isogenic *ompR* mutant and compared its phenotypic traits (bacterial growth, motility, biofilm formation, tolerance to food-relevant stresses, and virulence) with those of the wild-type strain. This work contributes to the understanding of factors governing STm survival and pathogenicity and provides insights for developing targeted control strategies against this pathogen in future research.

## 2. Materials and Methods

### 2.1. Bacterial Strains and Growth Conditions

Bacterial strains and plasmids used in this work are described in Table 1. *Salmonella* Typhimurium monophasic variant (STm) MRL040243, isolated from frozen pork, was used as the wild-type strain. This pathogen and *Escherichia coli* DH5α were stored in Luria/Bertani (LB) broth supplemented with 50% glycerol prior to use. The wild-type strain was streaked on Xylose Lysine Tergitol-4 (XLT4) agar plates and incubated at 37 °C overnight, followed by two successive subcultures for subsequent experiments.

### 2.2. Construction of the ompR Knockout Strain

#### 2.2.1. Plasmid Construction and Transformation

Primers used for mutant construction are listed in Table 2. Primers Apr-F/R and pKD46N-F/R were designed to amplify the apramycin resistance (Apr^r^) cassette from pIB139-EGFP and the pKD46 vector backbone lacking the Amp^r^ marker, respectively. These two fragments were assembled using a seamless cloning kit (Beyotime Biotechnology, Shanghai, China) to generate pKD46-Apr. Similarly, primer pair pCP20-Apr-F/R was used to amplify the Apr^r^ cassette from pKD46-Apr. The resulting PCR product was ligated into BamH I/PstI-double-digested pCP20 to yield pCP20-Apr. Purified pKD46-Apr was electroporated into the wild-type strain, and positive transformants were selected on apramycin-containing agar plates and verified by PCR and gene sequencing.

#### 2.2.2. Red-Mediated Homologous Recombination

Competent cells were prepared by culturing MRL040243 (pKD46-Apr) to an OD_600_ of 0.3, inducing λ Red recombinase with 30 mM L-arabinose, and then washing the cell pellet with 10% glycerol. A chloramphenicol resistance (Cm^r^) cassette flanked by *ompR* homology arms (amplified from pKD3 using primer pair *ompR*-Cm-F/R) was electroporated into the induced competent cells. Double-resistant (Cm^r^, Apr^r^) recombinants were screened after 72 h of incubation and verified by PCR and gene sequencing.

#### 2.2.3. Plasmid Elimination and FLP Recombination

Double-resistant clones were cultured at 41 °C to eliminate pKD46-Apr, yielding Cm^r^, Apr^r^ intermediate strains (Δ*ompR::Cm*). FLP recombinase was then expressed to excise the Cm resistance cassette, generating marker-free deletion mutant (Δ*ompR*), which were validated by PCR and gene sequencing.

### 2.3. Growth Kinetic Analysis

Single colonies of each strain were picked with sterile inoculation loops and cultured with shaking at 37 °C in LB broth. Bacterial viability was quantified at 2 h intervals through colony counting. The growth capacities of the wild-type (*wt*) and Δ*ompR* mutant strains were compared.

### 2.4. Swimming and Swarming Motility Assays

For swimming motility assays, the wild-type and mutant strains were cultured to stationary phase and adjusted to a concentration of 10^7^ CFU/mL. A 1.5 μL aliquot of each bacterial suspension was stab-inoculated into 0.3% LB agar plates (25 g/L LB broth, 3 g/L agar), avoiding contact with the plate bottom. After 5–10 min equilibration, plates were incubated at 37 °C for 9 h. Swimming halo diameters were measured in biological triplicate, and average values were used to compare swimming capacity between the strains.

For swarming motility assays, bacterial preparations followed the same protocol, with 1.5 μL suspensions spotted onto 0.5% LB agar plates (5 g/L LB broth, 5 g/L agar, 0.5% glucose). Plates were incubated at 37 °C for 11 h. Swarming halo diameters were measured in triplicate, and average values were used to compare swarming ability between the strains.

### 2.5. Biofilm Formation Assay

Biofilm-forming ability of the wild-type and mutant strains was quantified using the crystal violet staining method [28]. Stationary-phase bacterial cultures (approximately 10^9^ CFU/mL) were serially diluted in LB broth to 10^7^ CFU/mL, and 200 μL aliquots were transferred into 96-well plates, with sterile LB broth included as a blank negative control. After 48 h incubation at 37 °C, wells were gently washed with phosphate-buffered saline (PBS), air-dried, and stained with 1% crystal violet for 20 min. Following repeated PBS washes and complete drying, crystal violet bound to biofilms was solubilized in 200 μL per well of 95% ethanol, and absorbance was recorded at OD_580_. Biofilm formation phenotypes were categorized as none (OD_580_ < ODc), weak (ODc < OD_580_ ≤ 2ODc), moderate (2ODc < OD_580_ ≤ 4ODc), and strong (OD_580_ > 4ODc), where ODc represents the OD value of the negative control.

### 2.6. Thermal Stress Survival Assay

Stationary-phase cultures of the wild-type and mutant strains (approximately 10^9^ CFU/mL) were diluted 1:10 in LB broth and subjected to heat shock at 50 °C, 55 °C and 60 °C for 5 min and 10 min, respectively. All samples were then immediately cooled in an ice bath for 3 min. After treatment, the residual culturability of bacterial cells was determined by plating serially diluted cultures on LB agar plates.

### 2.7. Osmotic Stress Survival Assay

Stationary-phase cultures of the wild-type and mutant strains (approximately 10^9^ CFU/mL) were diluted 1:10 in LB broth supplemented with 10%, 20%, or 30% NaCl to evaluate bacterial survival under osmotic stress. After incubation for 2, 4, 6 days, the residual culturability of bacteria at each time point was determined by plating counting on LB agar.

### 2.8. Acid Stress Survival Assay

Stationary-phase cultures of the wild-type and mutant strains (approximately 10^9^ CFU/mL) were diluted 1:10 in HCl -adjusted LB broth with pH values ranging from 1 to 5 (pH = 1, 2, 3, 4, 5). LB broth at pH = 7.0 was used as the neutral control. All samples were incubated at 37 °C. After incubation for 1, 2, and 3 h, the residual culturability of bacteria was determined by plating counting on LB agar at each time point.

### 2.9. Oxidative Stress Survival Assay

Stationary-phase cultures of the wild-type and mutant strains (approximately 10^9^ CFU/mL) were exposed to sub-lethal concentrations (1 mM and 2 mM) and a lethal concentration (5 mM) of H_2_O_2_ in LB broth at 37 °C for 30 min and 60 min. After treatment, the residual culturability of bacteria was determined by plating counting on LB agar at each time point.

### 2.10. Antimicrobial Susceptibility Testing

According to the Clinical Laboratory Standardization Institute (M100, 2021) guidelines, the antimicrobial susceptibility of the wild-type and mutant strains was evaluated using the Kirby–Bauer disk diffusion method. Bacterial cultures at the stationary growth phase were collected and uniformly inoculated onto Mueller–Hinton (MH) agar plates in a cross-streak manner. The spreading procedure was repeated several times until the bacterial suspension was evenly distributed across the plate surface. Antibiotic susceptibility disks were then placed onto the inoculated MH agar plates using sterile forceps and gently pressed to ensure close contact between the disks and the agar surface. Three parallel replicates were prepared for each group, with disks evenly spaced from one another. The plates were inverted and incubated at 37 °C in a constant temperature and humidity incubator for 24 h, after which the inhibition zones were observed and recorded.

*E. coli* ATCC 25922 was used as the quality control strain. The concentrations of the antibiotic disks were as follows: PIP, Piperacillin (100 μg per tablet). AXO, Ceftriaxone (30 μg per tablet). FEP, Cefepime (30 μg per tablet). FAZ, Cefazolin (30 μg per tablet). CTX, Cefotaxime (30 μg per tablet). NIT, Furazolidone (300 μg per tablet). TAZ, Cefotiam (100 μg–10 μg per tablet). MERO, Meropenem (10 μg per tablet). SXT, Compound sulfonamide (25 μg per tablet). CIP, Ciprofloxacin (5 μg per tablet). TGC, Tigecycline (15 μg per tablet). IMI, Imipenem (10 μg per tablet). DOR, Doripenem (10 μg per tablet). GEN, Gentamicin (10 μg per tablet). ETP, Ertapenem (10 μg per tablet). MIN, Minocycline (30 μg per tablet). TET, Tetracycline (30 μg per tablet). LEVO, Levofloxacin (5 μg per tablet). TOB, Tobramycin (10 μg per tablet). AZT, Amikacin (30 μg per tablet). AMP, Ampicillin (10 μg per tablet). After incubation at 37 °C for 24 h, inhibition zone diameters were measured and interpreted as resistant (R), intermediate (I), or susceptible (S) according to CLSI recommended breakpoints.

### 2.11. Pathogenicity Assay

#### 2.11.1. Adhesion and Invasion Assay

Adhesion and invasion assays in Caco-2 cells were performed according to previously reported methods [29]. The bacterial suspension was resuspended after centrifugation, and the initial inoculum quantity was counted and recorded as *N*_0_. When the confluence of Caco-2 monolayer cells in 24-well plates reached 80%, the adhesion and invasion assays were conducted. Ten microliters of resuspended bacterial suspension was added, followed by infection of the cells at 37 °C for 1 h. After two rounds of washing with sterile 1× PBS, the cells were treated with 0.5 mL 0.25% trypsin and 0.5 mL 0.1% Triton X-100 for 3 min to achieve lysis. Finally, 20 μL of the resuspended bacterial solution was serially diluted 10-fold was spread onto LB agar plates, and bacterial counts were determined and recorded as *N*_1_. For the invasion assay, cells were infected for 2 h at 37 °C, and extracellular bacteria were eliminated via ertapenem sodium treatment, and intracellular bacteria were recovered by lysing the cells following the same procedure. The number of intracellular invasive bacteria was counted as *N*_2_. The adhesion and invasion rates were calculated using the following formulas:

Adhesion rate = *N*_1_/*N*_0_ × 100%, (1)

Invasion rate = *N*_2_/*N*_0_ × 100% (2)

#### 2.11.2. Experimental Animal Model

Four-to five-week-old ICR mice (Institute of Cancer Research) were purchased from Shanghai Jieshijie Laboratory Animal Co., Ltd., Shanghai, China. Mice were randomly allocated into experimental groups (n = 6 per group) and maintained under standard conditions with sterile food and water on time. All procedures were approved by the Medical Ethics Committee of Gongli Hospital, Pudong New Area, Shanghai, China (Approval date: 5 March 2025). Humane endpoints were strictly implemented throughout the animal experiment. Animals presenting with obvious lethargy, reduced food and water intake, body weight loss exceeding 20% of baseline weight, or severe disease-related clinical manifestations were immediately humanely euthanized in strict accordance with institutional IACUC policies to minimize animal suffering.

To simulate natural infection, 4-h-fasted ICR mice were gavaged with bacterial suspensions prepared from the wild-type or Δ*ompR* strains. The infection dosage was modified with reference to relevant published studies [30], and the total challenge dose for each animal was 1.28 × 10^11^ CFU. Control mice received an equivalent volume of sterile saline. Mice were monitored every 12 h for 7 days, with survival rates recorded daily. The lethal time for 50% mortality (LT_50_) was calculated from survival curves to evaluate virulence attenuation following *ompR* deletion in STm. Body weights were recorded daily for 7 days post-infection to assess infection-associated physiological changes.

Four-to five-week-old ICR mice (n = 6/group) were orally infected with the wild-type or mutant strains. At 48 h post-infection, liver, spleen, and intestinal tissues were collected, homogenized with sterile steel beads in 1 mL of sterile saline, and serially diluted in PBS buffer. Blood samples were obtained via retro-orbital bleeding. Bacterial loads were quantified by plating dilutions on *Salmonella*-selective agar (37 °C, 24 h) and enumerating CFUs.

Liver, spleen and intestinal tissues from infected mice (48 h post-infection) were fixed, dehydrated through graded ethanol, paraffin-embedded, and sectioned. After hematoxylin–eosin (HE) staining, pathological changes were examined by light microscopy.

### 2.12. Transcriptomic Analysis

*Salmonella wt* and Δ*ompR* strains were cultured overnight in LB broth, then diluted 1:100 with fresh LB broth and cultured until the mid-log phase. Subsequently, 10 mL of the bacterial culture was centrifuged at 4000 rpm for 20 min to collect the cells, which were washed three times with PBS and then frozen in liquid nitrogen for storage. RNA sequencing analysis was performed by Shanghai Winnerbio Technology Co., Ltd. (Shanghai, China). The procedure was as follows: Total RNA was extracted from the bacteria using TRIzol^®^ reagent (cat. no. 15596-026, Thermo Fisher Scientific Inc., Shanghai, China) and quantified. High-quality RNA samples (OD_260_/_280_ = 1.8–2.0, OD_260_/_230_ ≥ 2.0, RIN ≥ 8.0, 23S:16S ≥ 1.0, concentration ≥ 100 ng/μL, total amount ≥ 2 μg) were used for subsequent library construction. Ribosomal RNA was removed using the Ribo-clean rRNA Depletion Kit Mega (CAT. NO. RN417, Vazyme Biotech Co., Ltd., Nanjing, China), and a strand-specific RNA library was constructed using the VAHTS Universal V10 RNA-seq Library Prep Kit (CAT. NO. NR616, Vazyme Biotech Co., Ltd., Nanjing, China). The transcriptome library was sequenced using DNBSEQ-T7 platform. All samples passed sequencing quality control. The raw reads ≥ 27.48 M pairs, with Raw Q20 ≥ 96.30% and Raw Q30 ≥ 94.05%. After filtering, clean reads ≥ 27.38 M pairs, with Clean Q20 ≥ 96.42%, Clean Q30 ≥ 94.16%, and GC content ≥ 51.43%. The data were suitable for subsequent analysis. The mapping rate (Mapped_Ratio) of reads to the reference genome was ≥ 95.93% for all samples, indicating good alignment between the sequencing data and the reference genome.

The reference genome used in this study was derived from STm MRL040243. Gene expression levels were normalized using TPM and FPKM. Differential expression analysis was conducted with DESeq2 software (V1.38.3). The Benjamini–Hochberg (BH) method was applied for multiple testing correction. Genes with adjusted *p*-value ≤ 0.05, FDR ≤ 0.05 and fold change ≥ 2 were identified as differentially expressed genes. All raw transcriptome data from this study have been deposited in the NCBI Sequence Read Archive (SRA) under accession number PRJNA1476119.

### 2.13. Quantitative Real-Time PCR (RT-qPCR) Analysis

Total RNA was extracted from STm using the TRIzol^®^ reagent, followed by reverse transcription into cDNA using the Evo M-MLV Reverse Transcription Premix Kit (with gDNA Removal Reagent, Accurate Biology Co., Ltd., Changsha, China). Primers were designed using the Primer Premier 5.0 software and synthesized by Sangon Biotech Co., Ltd. (Shanghai, China). Subsequently, PCR reactions were carried out using the primers listed in Table S1 with the following programs: 1 cycle at 95 °C for 30 s, 40 cycles of 95 °C for 5 s and 60 °C for 30 s. A melt curve analysis was then conducted with the temperature profiles: 95 °C for 10 s, 65 °C for 60 s, and 97 °C for 1 s, at a heating ramp rate of 4 °C/s. The RT-qPCR analysis was performed using a real-time qPCR system (FQD-96A, BIOER, Hangzhou, China). Relative gene expression levels between the mutant and wild-type strains were determined using the 2^−∆∆Ct^ method, with 16S rRNA serving as the internal reference gene.

### 2.14. Statistics Analysis

All experiments were performed twice, with three replicates per experiment, and data are expressed as the mean ± standard deviation (SD). Statistical analyses were conducted using IBM SPSS Statistics 25.0, employing one-way ANOVA for multi-group comparisons and Student’s *t*-test for pairwise comparisons. GraphPad Prism 10.0 was used for data visualization. Significance levels were denoted as *p* < 0.05, *p* < 0.01, *p* < 0.001, and *p* < 0.0001.

## 3. Results

### 3.1. Construction and Growth Characterization of the ompR Mutant

**Figure 1:**
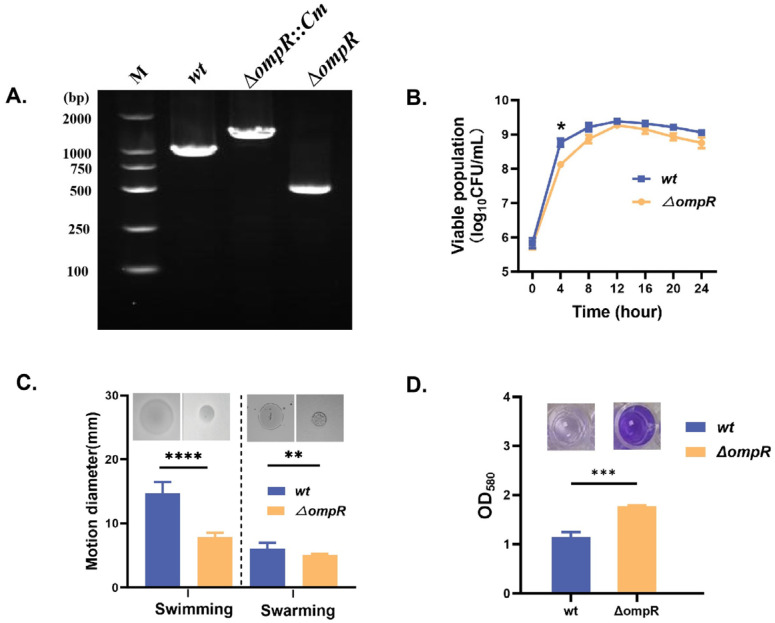
(**A**) Construction and PCR validation of the *ompR* mutant. The wild-type (*wt*), the first recombinant strain (Δ*ompR::Cm*), and the *ompR* knockout (Δ*ompR*). (**B**) Growth curves of the *wt* and Δ*ompR* mutant in LB broth. (**C**) Motility analysis of the *wt* and Δ*ompR* mutant. (**D**) Biofilm formation ability of the *wt* and Δ*ompR* mutant. Each data represents the mean ± standard deviation of at least three biological replicates. Asterisks represent significant differences (* *p* < 0.05, ** *p* < 0.01, *** *p* < 0.001, **** *p* < 0.0001).

Ampicillin resistance in plasmids pKD46 and pCP20 was successfully modified to apramycin resistance, and the corresponding plasmid profiles are shown in Supplementary Figures S1 and S2. To validate the *ompR* knockout in STm MRL040243, PCR verification was performed on three strains: the wild-type (*wt*), the first recombinant strain (∆*ompR:*:Cm), and the second recombinant strain (∆*ompR*). Genomic DNA was extracted from each strain and amplified using the primer pair *ompR*-F/*ompR*-R. The PCR products were analyzed via agarose gel electrophoresis to confirm knockout efficiency based on amplicon sizes (Table 1). The observed fragment lengths were compared to determine successful deletion of *ompR* in the recombinant strains (Figure 1A).

Growth curve analysis revealed synchronous growth patterns of the wild-type and ∆*ompR* strains in LB broth when initially inoculated at 5.8 log_10_ CFU/mL (Figure 1B). The ∆*ompR* mutant exhibited lower bacterial counts than the wild-type strain during the logarithmic growth phase (2–6 h), with a significant decrease in growth at 4 h (*p* < 0.05). By contrast, and both strains achieved comparable bacterial counts in the stationary phase (*p* > 0.05), with the wild-type strain peaking at 9.42 log_10_ CFU/mL and the ∆*ompR* mutant reaching 9.27 log_10_ CFU/mL. These findings suggest that deletion of *ompR* impairs bacterial growth during the logarithmic phase but has no obvious impact on the maximum biomass in the stationary phase.

### 3.2. OmpR Deletion Reduced Motility but Enhanced Biofilm Formation

Motility analysis revealed significant differences between the wild-type and ∆*ompR* mutant strains (Figure 1C). The wild-type strain exhibited an average swimming diameter of 14.7 mm, whereas the ∆*ompR* mutant showed a markedly reduced diameter of 7.87 mm, representing a 46.46% decrease in motility (*p* < 0.0001). Swarming ability analysis demonstrated similar trends, with the ∆*ompR* mutant forming smaller clusters (5.08 mm diameter) compared to the wild-type strain (6.03 mm), corresponding to an 18.70% reduction (*p* < 0.01). These findings collectively demonstrate that *ompR* deletion significantly reduces motility in STm.

As shown in Figure 1D, according to the biofilm classification criteria using calculated threshold values (ODc = 0.359, 2ODc = 0.718, 4ODc = 1.436), the ∆*ompR* mutant displayed strong biofilm-forming ability, whereas the wild-type strain only showed moderate biofilm formation. Statistical analysis confirmed biofilm production was significantly enhanced in the ∆*ompR* mutant relative to the wild-type strain (*p* < 0.001). Qualitative observation via crystal violet staining (Figure 1D) further validated this phenotypic difference. These findings indicate that *ompR* deletion markedly increases biofilm production.

### 3.3. OmpR Enhanced Environmental Stress Tolerance

#### 3.3.1. Thermal Tolerance

**Figure 2:**
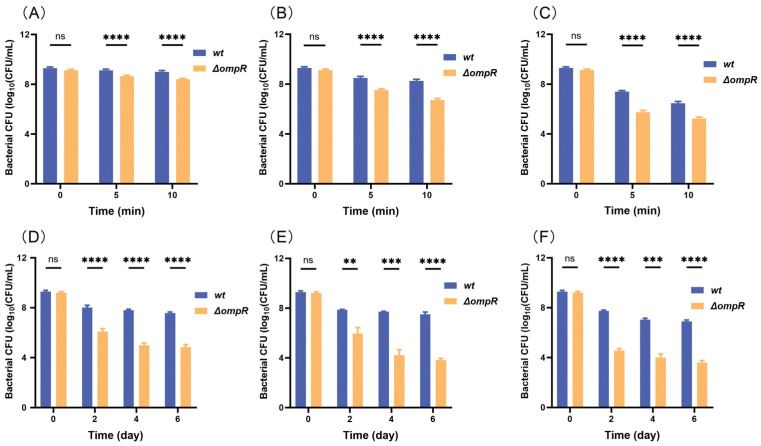
Tolerance of the wild-type and mutant strains to thermal and osmotic stress. (**A**–**C**) under different thermal treatment conditions, i.e., 50 °C, 55 °C, 60 °C. (**D**–**F**) under different osmotic treatment conditions, i.e., 10% NaCl, 20% NaCl, 30% NaCl. Asterisks represent significant differences (ns > 0.05, ** *p* < 0.01, *** *p* < 0.001, **** *p* < 0.0001).

Figure 2A–C show the survival of the wild-type and ∆*ompR* mutant strains after different heat treatments. Compared with the wild-type strain, the ∆*ompR* mutant displayed significantly lower viability (*p* < 0.0001) under identical treatment conditions. After treatment at 50 °C for 5 min, the mutant strain showed a reduction of 0.46 log_10_ CFU/mL. When the treatment time was extended to 10 min, the mutant strain exhibited a reduction of 0.7 log_10_ CFU/mL, whereas the wild-type strain showed only a 0.28 log_10_ CFU/mL reduction. Similarly, after treatment at 55 °C for 5 min, the mutant strain decreased by 1.6 log_10_ CFU/mL, while the wild-type strain decreased by only 0.79 log_10_ CFU/mL. Upon further exposure to 55 °C for 10 min, the reduction in the mutant strain was approximately twofold greater than that observed in the wild-type strain, with an extremely significant difference (*p* < 0.0001). A similar trend was also observed under treatment at 60 °C. These observations suggest that the absence of *ompR* may impair the capacity of STm to withstand thermal stress.

#### 3.3.2. Osmotic Stress Tolerance

Figure 2D–F present the survival of the wild-type and mutant strains under varying osmotic conditions. The ∆*ompR* mutant displayed significantly lower viability relative to the wild-type strain under identical treatment conditions (10%, 20%, and 30% NaCl). The above phenotypic differences imply that *ompR* deletion is likely to attenuate the osmotic stress resistance of STm.

#### 3.3.3. Acid Stress Tolerance

**Figure 3:**
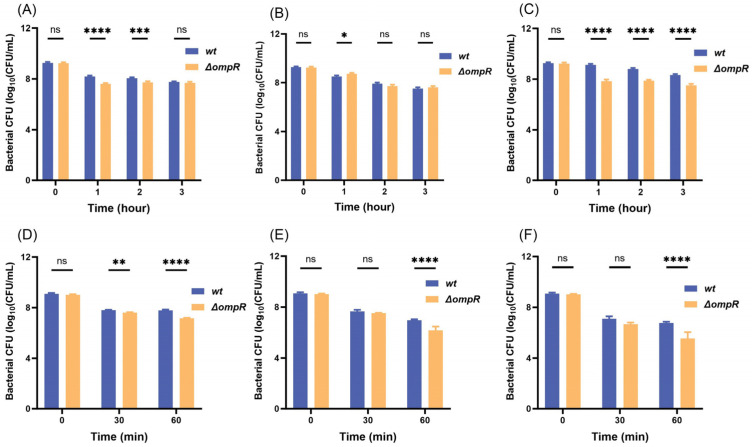
Tolerance of the wild-type and mutant strains to acid and hydrogen peroxide stress. (**A**–**C**) under different acid treatment conditions, i.e., pH = 3, pH = 4, pH = 5. (**D**–**F**) under different hydrogen peroxide treatment conditions, i.e., 1 mM, 2 mM, 5 mM. Asterisks represent significant differences (* *p* < 0.05, ** *p* < 0.01, *** *p* < 0.001, **** *p* < 0.0001).

The acid tolerance of the wild-type strain and the ∆*ompR* mutant is shown in Figure 3A–C. Under most acidic conditions, the mutant yielded significantly lower CFU counts than the wild-type strain (*p* < 0.001), indicating a greater reduction in bacterial counts.

No colonies of either strain were detected at pH = 1. At pH = 2, the wild-type strain exhibited a markedly higher number of viable colonies than the ∆*ompR* mutant strain (*p* < 0.0001) throughout the treatment. At pH = 3, the wild-type strain showed significantly higher number of viable colonies than the ∆*ompR* mutant during the first 2 h (*p* < 0.001). At pH = 4, the ∆*ompR* mutant initially exhibited a higher number of viable colonies than the wild-type strain within 1 h (*p* < 0.01), but subsequently showed significantly lower number of viable colonies (*p* < 0.01). At pH = 5, the CFU counts of the wild-type strain were significantly higher than that of the ∆*ompR* mutant (*p* < 0.0001) during the entire treatment period. Taken together, these phenotypic data suggest that loss of *ompR* may weaken the acid stress tolerance of STm.

#### 3.3.4. Hydrogen Peroxide Stress Tolerance

The tolerance of the wild-type and ∆*ompR* mutant strains to hydrogen peroxide stress is shown in Figure 3D–F. Under identical treatment conditions, the CFU counts of the ∆*ompR* mutant decreased more rapidly than those of the wild type, and the differences between the two strains increased over time (*p* < 0.05). Under 2 mM and 5 mM treatments, no obvious difference was observed within the first 30 min, while the ∆*ompR* mutant exhibited markedly reduced CFU counts after 60 min (*p* < 0.0001). Combined, these results raise the possibility that loss of *ompR* is associated with impaired survival and oxidative stress tolerance of STm upon hydrogen peroxide exposure.

### 3.4. Antibiotic Resistance

**Figure 4:**
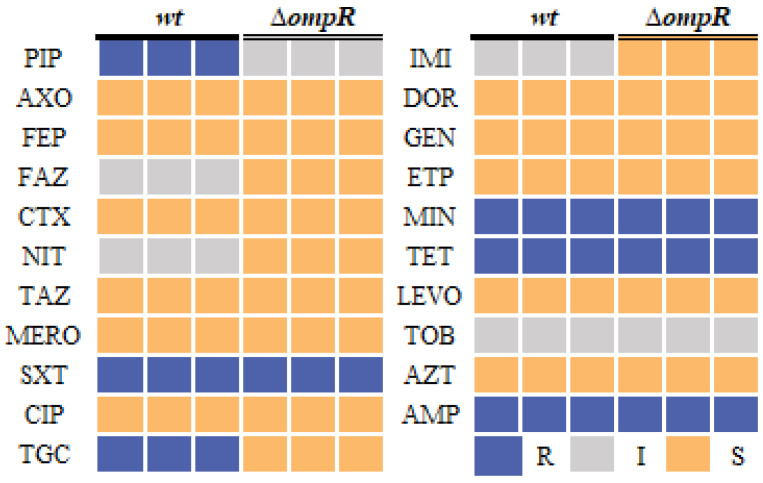
Antibiotic resistance of the wild-type (*wt*) strain and Δ*ompR* mutant. R, resistance. I, intermediate. S, susceptibility. PIP, Piperacillin. AXO, Ceftriaxone. FEP, Cefepime. FAZ, Cefazolin. CTX, Cefotaxime. NIT, Furazolidone. TAZ, Cefotiam. MERO, Meropenem. SXT, Compound sulfonamide. CIP, Ciprofloxacin. TGC, Tigecycline. IMI, Imipenem. DOR, Doripenem. GEN, Gentamicin. ETP, Ertapenem. MIN, Minocycline. TET, Tetracycline. LEVO, Levofloxacin. TOB, Tobramycin. AZT, Amikacin. AMP, Ampicillin.

The antimicrobial susceptibility profiles of the wild-type and ∆*ompR* strains against 21 antimicrobial agents are presented in Figure 4, with results classified as susceptible (S), intermediate (I), or resistant (R). As shown in Figure 4, the wild-type strain exhibited susceptibility to ceftriaxone, cefepime, cefotaxime, cefotiam, and 11 other antimicrobial agents, while demonstrating resistance to trimethoprim–sulfamethoxazole, minocycline, tetracycline, and ampicillin.

Notably, the ∆*ompR* mutant showed altered susceptibility to five antibiotics (i.e., piperacillin, cefazolin, furazolidone, tigecycline, and imipenem), with its susceptibility shifting from resistant to intermediate or from intermediate to susceptible. These findings suggest that *ompR* deletion may correlate with attenuated antibiotic resistance of STm to these five agents. Drug concentrations were set with reference to antimicrobial susceptibility breakpoints, and the changes in minimum inhibitory concentration (MIC) of strains under drug exposure were detected, as summarized in Supplementary Table S2. Notably, the mutant strains exhibited reduced MIC values against cefazolin, piperacillin and furazolidone. Collectively, our observations point to a likely linkage between functional *ompR* and the antimicrobial susceptibility phenotype of STm.

### 3.5. Bacterial Adhesion and Invasion to Caco-2 Cells

The adhesion and invasion capacities of the wild-type and ∆*ompR* mutant strains were determined in this study. Significant differences in cellular invasion efficiency were observed between the two strains (Supplementary Figure S3). The average adhesion rate of the wild-type strain was 1.08%, while ∆*ompR* strains exhibited a significantly increased adhesion rate of 1.83%. In contrast, the invasion efficiency showed an opposite tendency. The invasion rate of the ∆*ompR* strains was 0.46%, which was significantly lower than that of the wild-type strain (1.48%). In summary, deletion of *ompR* is closely associated with altered adhesion and invasion characteristics of STm to Caco-2 cells.

### 3.6. Role of ompR in Bacterial Pathogenicity

**Figure 5:**
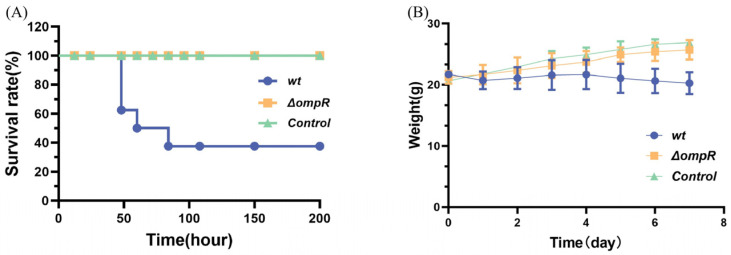
(**A**) Survival rates of ICR mice infected with each bacterial strain. (**B**) Body weight changes in each mouse group.

The clinical symptoms, body weight dynamics and survival rates of mice from different infection groups are shown in Figure 5. During the early infection stage (0–12 h), no apparent clinical signs were observed in infected mice, which maintained normal feeding and drinking activities, smooth fur, and regular locomotor activity. After approximately 12 h, abnormal changes began to appear, specifically manifested as motor dysfunction (e.g., circling behavior) and hyperactivity (e.g., frequent climbing of cage walls). By day 2, mice exhibited reduced activity, slowed responses, and mice often assumed curled-up or arched postures. Erect hair was observed on the body surface, fur became coarse and matted, eyelid secretions increased, some areas showed congestion or inflammation, eyes remained closed, and eventually death occurred. Mice inoculated with the ∆*ompR* mutant strain presented transient mild piloerection without any mortality, whereas mice in the control group maintained normal food intake and body weight with no abnormal clinical manifestations.

Body weight changes in each mouse group were plotted over time (Figure 5B). On day 7 post-infection, compared with the baseline at day 0, the ∆*ompR* mutant group had a weight gain of 21.26% with an average body weight of 25.71 g, while the control group achieved a 30.33% weight gain and an average body weight of 26.9 g, demonstrating slower body weight growth in mice infected with the mutant strain. In contrast, the wild-type group demonstrated progressive weight loss, declining by 6.74%.

Survival rates of ICR mice infected with each bacterial strain are presented (Figure 5A). Both the ∆*ompR* mutant group and control group maintained 100% survival rate during the whole observation period, whereas the wild-type strain caused mortality within 48 h post oral challenge. The 50% lethal time (LT_50_) of STm was further determined under a defined challenge dose (1.28 × 10^11^ CFU). The LT_50_ value of the wild-type strain was calculated as 72 h, while no death was recorded in either the ∆*ompR* mutant group or the control group. The virulence of the ∆*ompR* mutant is significantly attenuated in this mouse oral infection model (*p* < 0.01).

Intestinal histological lesions of mice from different groups are displayed in Supplementary Figure S4. Severe intestinal congestion and significant intestinal elongation were detected in wild-type-infected mice relative to Δ*ompR*-infected mice. Deletion of *ompR* contributes to virulence attenuation of STm in vivo, suggesting that this gene may participate in the virulence regulation of STm.

### 3.7. Bacterial Load Quantification in Mouse Liver, Spleen, Intestines, and Blood

**Figure 6:**
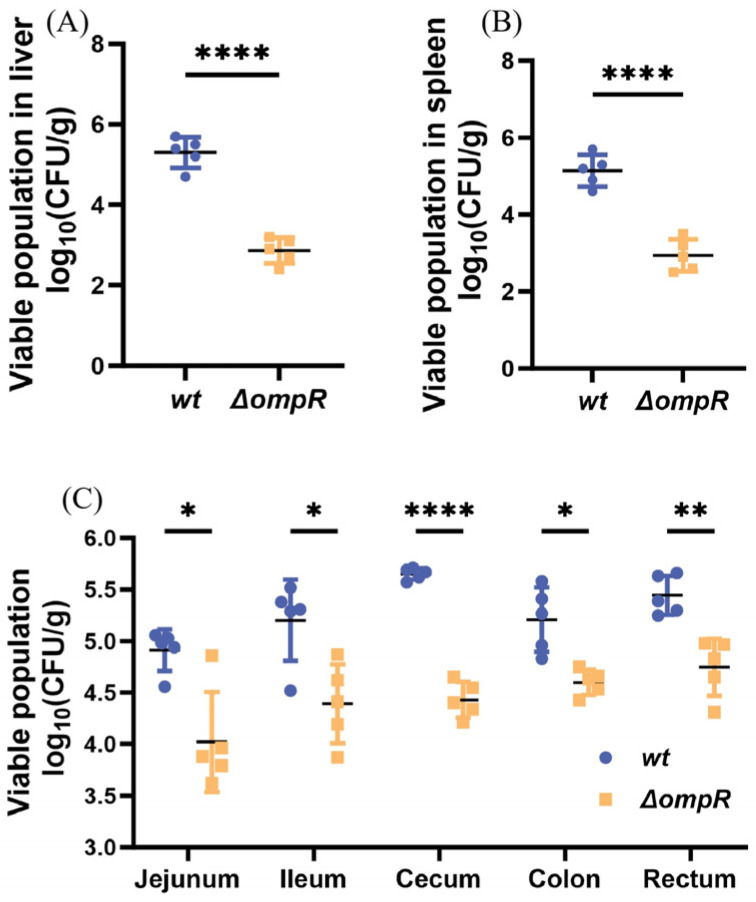
The results of bacterial load measurements in the liver (**A**), spleen (**B**), and intestines (**C**) of mice infected with the wild-type and Δ*ompR* mutant strains. Asterisks represent significant differences (* *p* < 0.05, ** *p* < 0.01, **** *p* < 0.0001).

Bacterial loads in the liver, spleen, and intestines of mice were quantified at 48 h post oral infection (Figure 6A–C). In the wild-type infection group, the bacterial loads in the liver and spleen were 5.30 log_10_ CFU/g and 5.14 log_10_ CFU/g, respectively. By comparison, significantly lower bacterial loads were detected in the ∆*ompR* group, with 2.86 log_10_ CFU/g in liver and 2.94 log_10_ CFU/g in spleen (Figure 6A,B). No bacteria were detected in the blood samples of mice infected with either the wild-type strain or the ∆*ompR* mutant.

After 48 h of infection, the bacterial loads in the jejunum, ileum, cecum, colon, and rectum of mice infected with the wild-type strain were as follows: 4.91 log_10_ CFU/g, 5.20 log_10_ CFU/g, 5.65 log_10_ CFU/g, 5.21 log_10_ CFU/g, and 5.45 log_10_ CFU/g, respectively (Figure 6C). After 48 h of infection, the bacterial counts in the jejunum, ileum, cecum, colon, and rectum of mice infected with the ∆*ompR* mutant were as follows: 4.02 log_10_ CFU/g, 4.39 log_10_ CFU/g, 4.43 log_10_ CFU/g, 4.60 log_10_ CFU/g, and 4.75 log_10_ CFU/g, respectively.

Compared with the wild-type strain, the ∆*ompR* mutant showed a significantly reduced bacterial load in the liver, spleen, and cecum of mice (*p* < 0.0001). These results indicate that *ompR* deletion is associated with impaired in vivo colonization ability of STm in mice.

### 3.8. Observation of Pathological Sections

Histopathological examination of the liver (Supplementary Figure S4) showed that the overall hepatic structure remained intact in both wild-type-infected and ∆*ompR*-infected groups. Livers from mice infected with the wild-type strain displayed normal tissue organization, with intact central vein structure (red arrow) and radially arranged hepatic sinusoids, without significant tissue edema, inflammatory infiltration, or structural loosening. In contrast, Δ*ompR*-infected livers exhibited moderate architectural abnormalities (yellow arrow), characterized by hepatocellular edema and tissue loosening, though maintaining regular cellular arrangement. Notably, neither strain induced significant vascular congestion, sinusoidal dilation, or inflammatory cell infiltration. These findings demonstrate that both the wild-type strain and the Δ*ompR* mutant induce only mild hepatic pathology following oral challenge, with no clinically relevant differences in lesion severity between the two strains.

Histopathologic analysis of the spleen (Supplementary Figure S5) revealed obvious differences among the three groups. The control group maintained normal splenic architecture, while the wild-type strain exhibited mild tissue abnormalities characterized by preserved splenic follicles (yellow arrows) without evident atrophy or necrosis, accompanied by focal macrophage proliferation (red arrows) and occasional multinucleated giant cell infiltration (black arrows). By contrast, the Δ*ompR* mutant displayed more apparent alterations, including follicular atrophy (yellow arrow) and prominent multinucleated giant cell infiltration (black arrow). Although both strains induced mild splenic pathology overall, lesion in the mutant group were more prominent than those in the wild-type group.

Histopathological analysis of the gastrointestinal tract (Supplementary Figure S6) showed distinct pathological patterns among the three groups. The wild-type strain induced significant intestinal damage, including jejunal villi edema (yellow arrows), mucosal layer separation (red arrows), and structural discontinuity (black arrows), while the jejunum of mice infected with the Δ*ompR* mutant maintained a nearly normal architecture. In ileal sections, the wild-type strain caused villi shortening and goblet cell reduction, whereas the Δ*ompR* group showed only focal epithelial desquamation (red arrow). Cecal examination demonstrated extensive mucosal necrosis in the wild-type group, compared to moderate epithelial abnormalities in the Δ*ompR* mutant group. Both colon and rectal sections confirmed milder pathology in the Δ*ompR* group, with limited epithelial damage and no submucosal edema, in contrast to the changes induced by the wild-type strain. In general, the two strains triggered tissue damage in a tissue-dependent manner: the ∆*ompR* mutant caused slightly more severe lesions in the liver and spleen, but markedly milder injury in the gastrointestinal tract. Overall, the ∆*ompR* mutant exhibited attenuated virulence in the mouse infection model.

### 3.9. Transcriptomic Profiles

**Figure 7:**
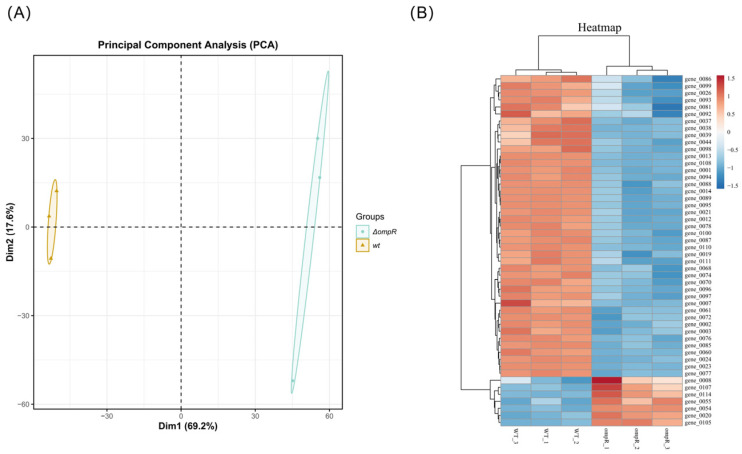
Sample correlation analysis: (**A**) PCA (Principal Component Analysis) and (**B**) Cluster Analysis.

**Figure 8:**
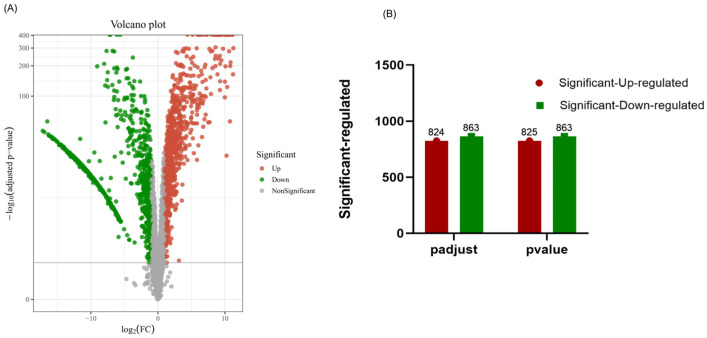
Gene expression changes at the transcriptional level. (**A**) The volcano plot visualizes the transcriptomic results, with red and green dots representing upregulated and downregulated genes, respectively. (**B**) A visualization of the number of upregulated and downregulated genes.

The transcriptomic profiles of the wild-type and mutant strains were revealed by RNA sequencing analysis. Principal component analysis (PCA) revealed a clear separation between the wild-type and ∆*ompR* mutant strains along PC1 (Figure 7A), and the clustering results showed a weak correlation (Figure 7B). These findings indicate that deletion of *ompR* markedly altered the global transcriptional profile of STm. Additionally, biological replicates clustered closely together, demonstrating high reproducibility and reliability of the RNA-seq data. The gene expression differences were analyzed between the *wt* and Δ*ompR* strains. The results showed that 824 genes were significantly upregulated and 863 genes were significantly downregulated in the Δ*ompR* strain compared to the wild-type strain (Figure 8A,B).

**Figure 9:**
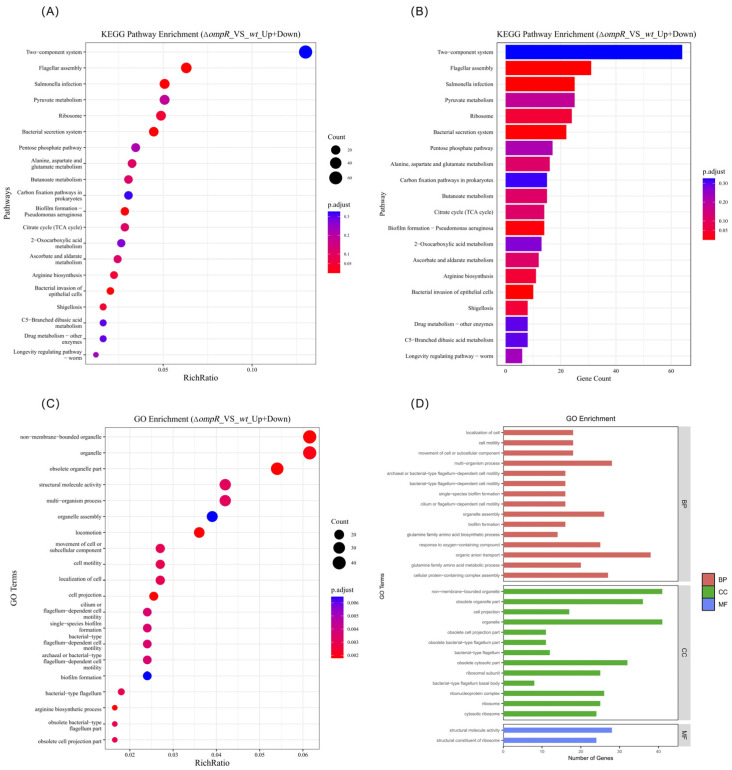
KEGG pathway enrichment analysis and GO enrichment analysis. (**A**) KEGG pathway enrichment bubble plot. (**B**) KEGG pathway enrichment bar plot. (**C**) GO term enrichment bubble plot. (**D**) GO term enrichment bar plot. Note: The vertical axis represents the pathway names, while the horizontal axis represents the Rich factor. The size of the points reflects the number of genes in the gene set annotated to the corresponding pathway, and the color of the points corresponds to different ranges of q-values. The Rich factor refers to the ratio of the number of differentially expressed genes annotated to the pathway (sample number) to the total number of genes annotated to the pathway (background number). A larger Rich factor indicates a higher degree of enrichment.

KEGG analysis showed that the differentially expressed genes were mainly associated with metabolic pathways (e.g., pyruvate metabolism and the citric acid cycle) as well as pathogenic mechanisms, including bacterial secretion systems, flagellar assembly, and two-component systems (Figure 9A,B). GO gene enrichment analysis revealed that the upregulated GO terms were categorized into three categories: Biological Process (16 terms, 55.2%), Cellular Component (2 terms, 6.9%), and Molecular Function (11 terms, 37.9%). In contrast, the downregulated GO terms were associated with resistance (10%), motility (30%), biofilm formation (6.7%), and other biological processes (Figure 9C,D).

Further analysis revealed that genes significantly upregulated in the ∆*ompR* mutant strain compared with the wild-type strain were mainly enriched in pili-related pathways (Table S3), which may be involved in the regulation of bacterial adhesion and biofilm formation. Meanwhile, the expression levels of genes associated with flagellar biosynthesis and virulence were significantly downregulated in the ∆*ompR* strain (Table S4), suggesting that this mutant defected in flagellar formation and abnormal virulence expression.

### 3.10. Validation of Differentially Expressed Genes by RT-qPCR

**Figure 10:**
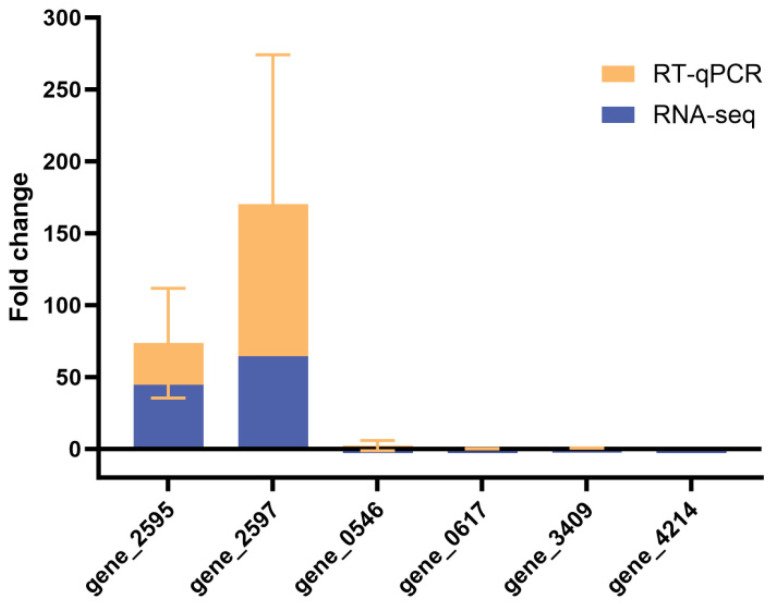
Comparison of gene expression patterns revealed by the RNA-seq and RT-qPCR tests.

Several differentially expressed genes were selected for RT-qPCR analysis, and their expression profiles obtained from RNA-seq and RT-qPCR was subsequently compared. As shown in Figure 10, the overall differential expression trends of gene_0546, gene_0617, gene_2595, gene_2597, gene_3409 and gene_4214 were consistent between the two methods. These results support that the RNA-seq experiment was appropriately performed and the generated data are reliable.

## 4. Discussion

STm has become a growing public health concern due to its widespread dissemination and enhanced resistance to environmental stresses [31]. Understanding the molecular mechanisms that underpin its adaptability and virulence is critical for the development of targeted prevention and control strategies. The EnvZ/OmpR two-component system is widely recognized for its role in regulating bacterial responses to environmental changes. However, effects of *ompR* on environmental tolerance and virulence in STm remain poorly characterized. In this study, an isogenic *ompR* deletion mutant was constructed, and phenotypic combined with transcriptomic analyses were performed to explore the function of OmpR in STm physiology and pathogenicity.

We first evaluated the influence of *ompR* deletion on bacterial motility. The Δ*ompR* mutant showed a significantly smaller swimming and swarming zones than the wild-type strain, demonstrating that loss of *ompR* impairs both types of motility. Interestingly, the function of *ompR* in motility regulation exhibits obvious species specificity. Previous studies reported that *ompR* deletion has no effect on motility in *E.coli* [32], while it remarkably inhibits motility in *Edwardsiella piscicida* [33]. It has been demonstrated that the EnvZ/OmpR system modulates the expression of flagellar and type I pili genes to regulate bacterial motility. Such differences reflect the functional divergence of *ompR* among different bacterial species.

Both crystal violet staining and quantitative biofilm assays verified that biofilm formation was significantly enhanced in the Δ*ompR* mutant relative to the wild-type strain (*p* < 0.001). A similar phenotype was observed in *Vibrio cholerae*, in which *ompA* acts as a negative regulator of biofilm formation [34]. On the contrary, research on *Aeromonas veronii* showed that *ompR* knockout suppressed biofilm formation [35]. Combined with the above findings, we hypothesize that OmpR potentially acts as a negative regulator of biofilm formation in STm. Further complementary assays including gene complementation strains are still required to validate this regulatory relationship in our future work.

Phenotypic stress tolerance assays showed that the Δ*ompR* mutant displayed impaired resistance to multiple adverse stresses, including heat, osmotic pressure, acid and hydrogen peroxide, indicating that OmpR participates in the stress adaptation of STm. It is worth noting that the data in this study were based on colony-forming unit (CFU) counts, which reflect the number of culturable viable bacteria within the population rather than the actual bacterial survival rate. Under stress conditions, a portion of bacterial cells may enter a viable but non-culturable (VBNC) state [36], which cannot be detected by CFU-based methods. Therefore, the results of this study represent only the population of culturable cells and may not fully reflect the total viable bacterial population.

Antibiotic susceptibility tests showed that *ompR* deletion did not alter the bacterial resistance against trimethoprim–sulfamethoxazole, minocycline, tetracycline and ampicillin. However, the Δ*ompR* mutant became more susceptible to five antibiotics (piperacillin, cefazolin, furazolidone, tigecycline and imipenem), with resistance phenotypes shifting from resistant to intermediate or intermediate to susceptible. The tests also displayed decreased MIC values when treated with cefazolin, piperacillin and furazolidone. These antibiotics mainly act on cell wall synthesis, ribosome function or DNA replication [37]. We preliminarily speculate that *ompR* may indirectly regulate bacterial membrane integrity, efflux systems or stress response pathways, thereby affecting susceptibility to these antimicrobials.

In vivo mouse infection experiments confirmed that *ompR* deletion significantly attenuated the pathogenicity of STm. The Δ*ompR* mutant failed to cause lethal infection in mice within the observation period, and the bacterial loads in mouse liver and spleen were significantly lower than those in the wild-type infection group. Histopathological analysis revealed that both strains caused mild lesions in the liver and spleen, while lesions in the Δ*ompR* group were slightly more severe. This finding is consistent with previous studies in *E. coli*, in which *ompR* deficiency impaired bacterial colonization in host organs [38]. The mild pathological changes in liver and spleen indicate that although STm can disseminate to visceral organs, severe tissue damage relies on sustained colonization and high bacterial loads, a process associated with OmpR.

Outer membrane usher proteins and structural proteins encoded by fimbrial biogenesis-related genes are responsible for the assembly of diverse fimbriae [39,40]. Type 1 fimbriae primarily mediate bacterial adhesion to host cells, and curli fimbriae are crucial for biofilm formation and bacterial aggregation [41]. In the present study, multiple fimbria-associated genes (*fimD*, *stiA*, *yehD* and *lpfA*) were significantly upregulated in the Δ*ompR* mutant. These genes are well-documented to regulate fimbrial assembly, adhesion and biofilm formation [42]. Therefore, we speculate that *ompR* deletion enhances biofilm formation of STm via upregulating the expression of fimbria-related genes.

In contrast to the upregulation of fimbria-related genes, *ompR* deletion also led to significant downregulation of multiple genes and pathways closely associated with bacterial virulence, environmental adaptation, and physiological metabolism. Specifically, the significantly downregulated pathways and genes shown in Table S3 are mainly involved in type III secretion system virulence effectors, oxygen-regulated invasion proteins, SPI-1 and SPI-2 type III secretion system effectors, as well as the transcriptional regulator HilA, all of which are closely associated with bacterial virulence [43,44]. This indicates that *ompR* may participate in regulating bacterial virulence by modulating the expression of these genes.

As a key signal transduction system in bacteria, the two-component system is also regulated by the *ompR* gene. Following *ompR* knockout, the expression levels of multiple porin genes (e.g., *ompD*, *ompC*), two-component system-related genes (e.g., response regulator *rstA*, sensor histidine kinases *ttrS*, *phoQ*, and *envZ*), lipoprotein and family protein genes (e.g., *mipA/ompV*, *ompA*), and transcriptional regulator genes (e.g., *sdiA*) were all significantly downregulated. Previous studies have confirmed that these aforementioned genes are important sensing and signal transduction elements that STm relies on for survival in harsh environments. This further indicates that two-component systems play an important role in the environmental stress response of STm and that *ompR* knockout may impair the environmental adaptability of STm by downregulating these genes.

In addition, the expression levels of multiple genes closely related to bacterial physiological functions were also significantly downregulated after *ompR* knockout. These included genes associated with multidrug resistance and efflux transport systems, genes related to the glutathione-regulated potassium efflux system (e.g., KefC, KefG), genes involved in antioxidant defense and substance transport (e.g., superoxide dismutase and catalase HPII), as well as response regulator genes (e.g., *phoB* and *ssrB*). These results suggest that *ompR* may play a role in regulating the overall physiological metabolism and environmental adaptability of STm.

Collectively, OmpR in STm plays a multifaceted role in regulating growth dynamics, motility, biofilm formation, stress response, antibiotic susceptibility, and virulence. Its deletion leads to pleiotropic effects that suggesting that it may occupy an important position in the regulatory network governing bacterial adaptation and pathogenicity.

## 5. Conclusions

This study successfully constructed an *ompR*-deleted strain of STm. The *ompR* deletion significantly enhanced biofilm formation capacity of STm. Furthermore, the mutant exhibited reduced tolerance to heat, osmotic pressure, acid, and oxidative stress. In vivo assays revealed that the mutant lost lethal virulence and had reduced organ colonization ability. Pathological changes varied among tissues: the mutant caused mild intestinal injury but slightly aggravated lesions in the liver and spleen. Collectively, these findings highlight the potential regulatory role of *ompR* in STm stress tolerance and virulence, providing valuable insights into the biological function of this two-component regulator in *Salmonella* pathogenicity.

## Figures and Tables

**Table 1:** Strains and plasmids used in this work.

Strains/Plasmids	Relevant Features
Strains	
STm MRL040243 (wild-type strain)	Resistant to ampicillin and kanamycin, susceptible to chloramphenicol and apramycin
*E. coli* DH5α	cloning host strain
Plasmids	
pKD46	Amp^r^, low-copy temperature-sensitive replicon oriR101; expresses recombinases of the λ Red recombination system
pKD3	Amp^r^, Cm^r^, carries FRT (FLP recombinase target) recognition sites
pCP20	Amp^r^, Cm^r^, low-copy temperature-sensitive replicons oriR101, expresses FLP recombinase that excises resistance cassettes by recognizing FRT sites
pIB139-EGFP	Apr^r^, carries apramycin resistance cassette for transformant screening
pKD46-Apr	Apr^r^, derivative of pKD46, with its ampicillin resistance marker replaced by an apramycin resistance cassette
pCP20-Apr	Cm^r^, Apr^r^, derivative of pCP20, with its ampicillin resistance marker replaced by an apramycin resistance cassette

**Table 2:** Primer sequences used for mutant construction.

Primer Name	Primer Sequence (5′–3′)
Apr-F	aatattgaaaaaggaagagtATGTCATCAGCGGTGGAGTGC
Apr-R	gagtaaacttggtctgacagTCAGCCAATCGACTGGCG
pKD46N-F	ACTCTTCCTTTTTCAATATTATTGAAGC
pKD46N-F	CTGTCAGACCAAGTTTACTCATATATACTTT
pCP20-Apr-F	acgttgttgccattgctgcagTCAGCCAATCGACTGGCG
pCP20-Apr-R	tgcgtccggcgtagaggatccCGCGGAACCCCTATTTGTT
*ompR*-F	TTGCGCACACGGGGTATAACG
*ompR*-R	CTGGAGGCTCGGCAAAATCG
*ompR*-Cm-F	atgcaagagaattataagattctggtggttgatgacgatatgcgtctgcgCCATATGAATATCCTCCTTAG
*ompR*-Cm-R	tcatgctttagaaccgtccggtacaaagacgtagcccaggccccagacggTGTAGGCTGGAGCTGCTTC

## Data Availability

The data of this study are available from the authors upon reasonable request.

## Supplementary Materials

The following supporting information can be downloaded at: https://www.mdpi.com/article/10.3390/microorganisms14071503/s1, Figure S1: pKD46-Apr plasmid map; Figure S2: pCP20-Apr plasmid map; Figure S3: Caco-2 cell adhesion and invasion assay; Figure S4: Morphological changes of intestinal histology; Figure S5: Mouse liver and spleen tissue observation by light microscopy; Figure S6: Mouse intestinal tissue observation by light microscopy; Table S1: Primers used for RT-qPCR analysis; Table S2: MIC variations of strains treated with antibiotics; Table S3: Upregulated genes after *ompR* deletion in STm strain; Table S4: Downregulated genes after *ompR* deletion in STm strain.
