# Supplementary material for: Effects of ompR Deletion on Stress Tolerance and Virulence in Salmonella Typhimurium Monophasic Variant
Source: Microorganisms. 2026 Jul 9;14(7):1503. doi: 10.3390/microorganisms14071503 (PMC13413605; doi:10.3390/microorganisms14071503)
Supplement: Supplementary file 1 [file microorganisms-14-01503-s001.zip › microorganisms-4326124-supplementary.pdf]

Supplementary Materials:

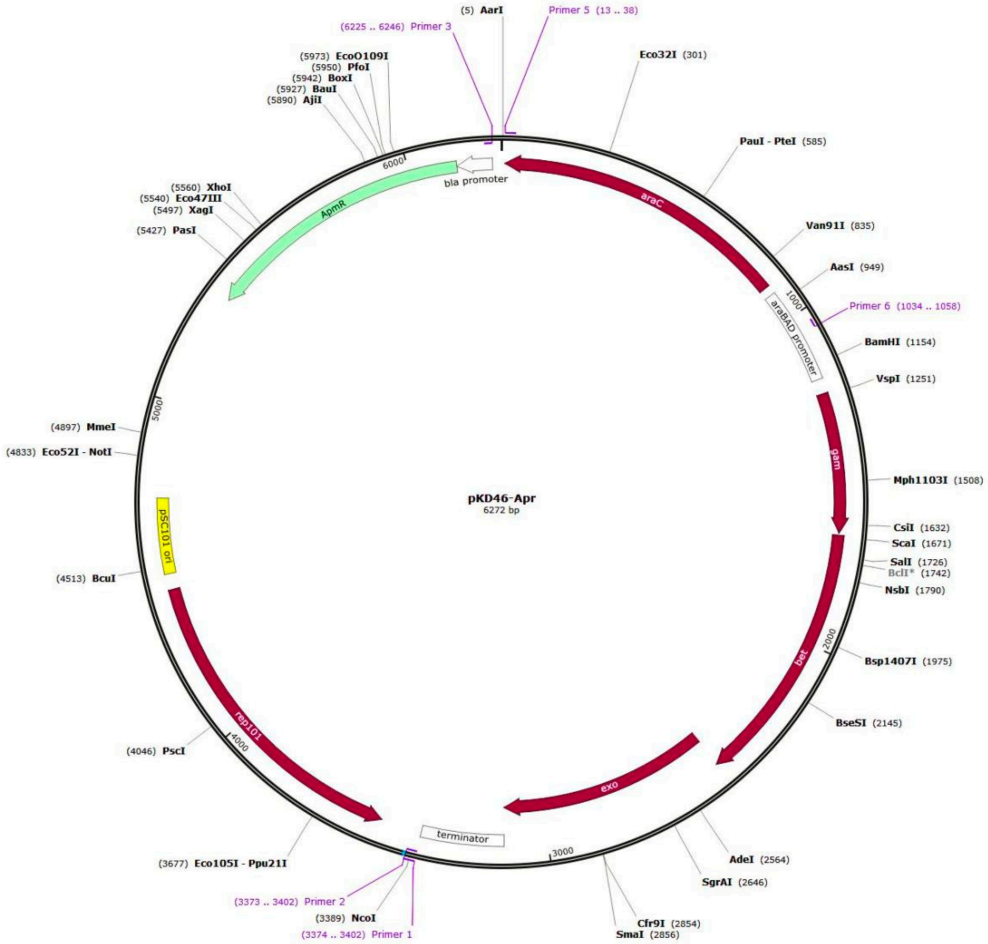

Figure S1 pKD46-Apr plasmid map.

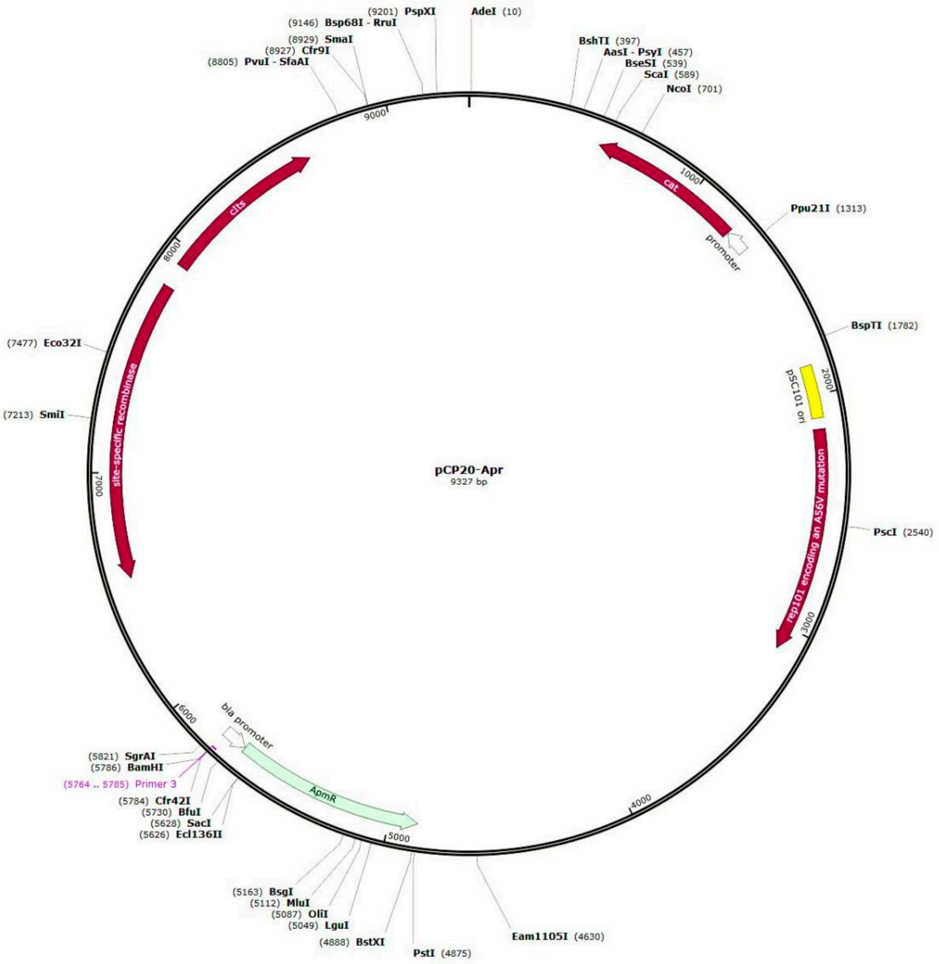

Figure S2 pCP20-Apr plasmid map.

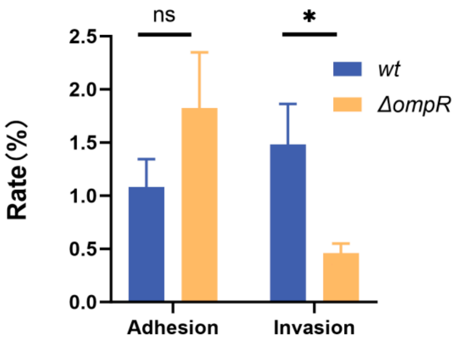

Figure S3 Caco-2 cell adhesion and invasion assay results (* $p < 0.05$).

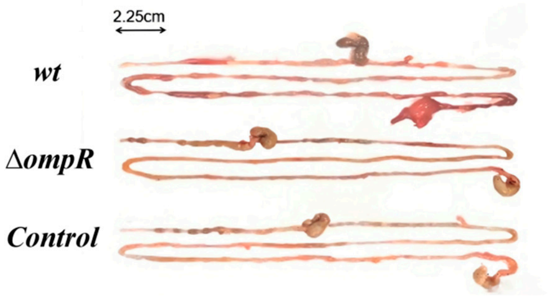

Figure S4 Morphological changes of intestinal histology.

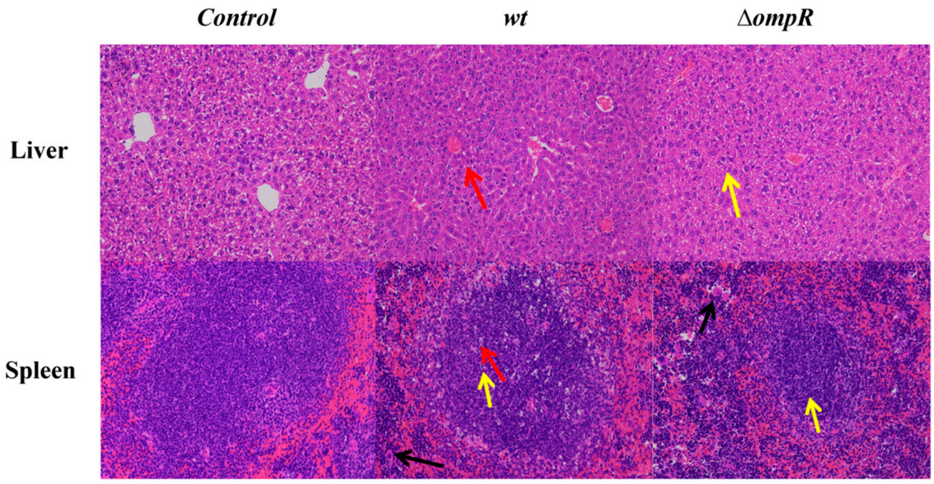

Figure S5 Mouse liver and spleen tissue observation by light microscopy.

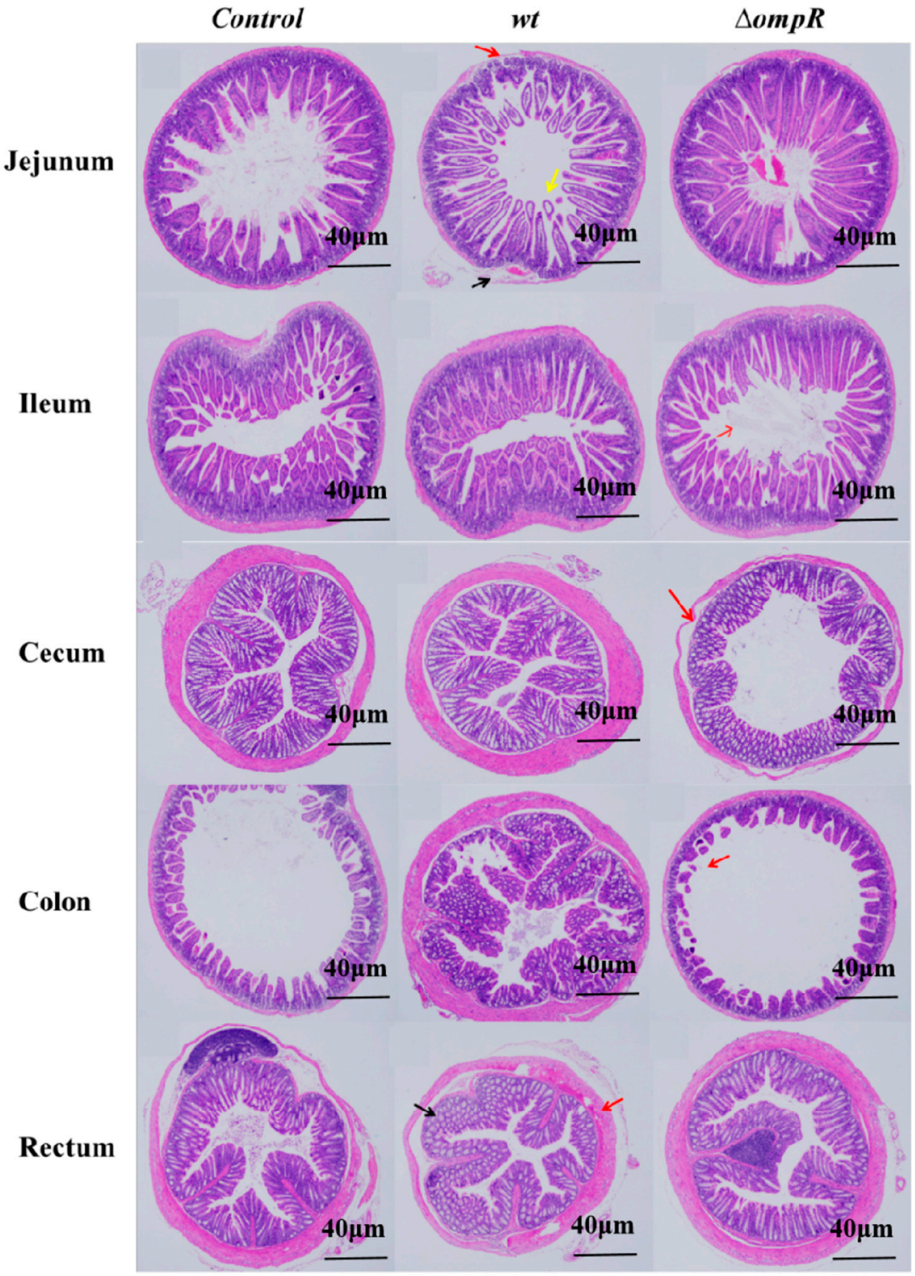

Figure S6 Mouse intestinal tissue observation by light microscopy.

Table S1. Primers used for RT-qPCR analysis.

Gene	Forward primer sequence (5′–3′)	Reverse primer sequence (5′–3′)	Primer size (bp)
gene_2595	ACAGGCAAGGTCAGCAAC	CGACGCCATTATTCACAG	169
gene_2597	GGTGAATCAGGGCGAGCAA	CTGGCGGCGTCAGCATAA	107
gene_4214	AGCCACGGTTTCTCAG	GGACGCTATTTCCTTTT	143
gene_0546	GCATACGAGCCTGACATA	AACATAGCCATTAGCACC	174
gene_0617	CCTTACAGCCTGAGACG	CCAGTGGAAACGGACA	149
gene_3409	GCGTGGTGGTCTTG	CTGCTCCAGTTTATCC	169
16 S rRNA	CAGAAGAAGCACCGGCTAAC	GACTCAAGCCTGCCAGTTTC	316

Table S2 MIC variations of strains treated with antibiotics

Piperacillin				Cefazolin			
**Strain** / **Concentration**	*wt*	*ΔompR*	*E. coli* ATCC 25922	**Strain** / **Concentration**	*wt*	*ΔompR*	*E. coli* ATCC 25922
8μg/ml	+	+	-	3μg/ml	+	+	-
16μg/ml	+	+	-	4μg/ml	+	+	-
32μg/ml	+	+	-	5μg/ml	+	+	-
64μg/ml	+	+	-	6μg/ml	+	+	-
128μg/ml	+	+	-	7μg/ml	+	+	-
256μg/ml	+	-	-	8μg/ml	+	-	-
Furazolidone							
4μg/ml	+	-	-				
8μg/ml	-	-	-				
16μg/ml	-	-	-				
32μg/ml	-	-	-				
64μg/ml	-	-	-				
128μg/ml	-	-	-				

Table S3 Upregulated genes after *ompR* deletion in STm strain

Gene_ID	*ompR*_VS_*wt*_Fold Change	*ompR*_VS_*wt*_log2FoldChange	Regulate	Gene_Type	NR_Annotation
gene_0344	25.34	4.66	Up	--	virulence effector SrfC
gene_1055	4.76	2.25	Up	*parE1_3_4*	type II toxin-antitoxin system RelE/ParE family toxin
gene_2385	2.07	1.05	Up	*relB*	type II toxin-antitoxin system antitoxin, RelB/DinJ family
gene_2595	44.58	5.48	Up	--	putative fimbrial-like adhesin protein
gene_1739	5.72	2.52	Up	--	long polar fimbrial protein LpfA
gene_2596	23.96	4.58	Up	*fimD*	fimbrial biogenesis outer membrane usher protein
gene_2597	64.28	6.01	Up	--	fimbrial assembly protein
gene_2598	175.11	7.45	Up	--	fimbrial protein YehD

Gene_ID	*ompR*_VS_*wt*_Fold Change	*ompR*_VS_*wt*_log2FoldChange	Regulate	Gene_Type	NR_Annotation
gene_2802	2.21	1.15	Up	--	fimbrial protein StiA
gene_0470	2.19	1.13	Up	*rstB*	two-component system sensor histidine kinase RstB
gene_2241	2.73	1.45	Up	*arcA*	two-component system response regulator ArcA
gene_4198	146.37	7.19	Up	*ompN*	outer membrane protein S1
gene_4582	149193.03	17.19	Up	*strA*	aminoglycoside O-phosphotransferase APH(3''-Ib
gene_4620	24102.24	14.56	Up	*blaTEM*	TEM family class A beta-lactamase
gene_3620	2.55	1.35	Up	*mdfA*	multidrug transporter MdfA
gene_0422	2.92	1.55	Up	*marA*	MDR efflux pump AcrAB transcriptional activator MarA
gene_0421	3.02	1.60	Up	*marR*	multiple antibiotic resistance transcriptional regulator MarR
gene_0676	2.53	1.34	Up	*ABC.PE.P1*	ABC transporter permease
gene_0677	2.34	1.23	Up	*ABC.PE.P*	ABC transporter permease
gene_4064	2.18	1.12	Up	*TC.MATE*	MATE family efflux transporter DinF
gene_4111	6.28	2.65	Up	*acrF*	efflux RND transporter permease subunit
gene_4112	13.41	3.75	Up	*acrE*	efflux RND transporter periplasmic adaptor subunit
gene_4245	2.18	1.12	Up	*kefB*	glutathione-regulated potassium-efflux system protein
gene_4445	9637.96	13.23	Up	*tetA*	tetracycline resistance protein, class B (TetA(B)) (Metal-tetracycline/H(+) antiporter)
gene_1263	7436.4	12.86	Up	*rpoE*	RNA polymerase subunit sigma-70
gene_0958	2.12	1.08	Up	*fhlA*	formate hydrogen-lyase transcriptional activator
gene_1347	2.41	1.27	Up	TC.DASS	DASS family sodium-coupled anion symporter

Table S4 Downregulated genes after *ompR* deletion in STm strain

Gene_ID	*ompR*_VS_*wt*_Fold Change	*ompR*_VS_*wt*_log2Fold Change	Regulate	Gene_Type	NR_Annotation
gene_0869	0.08	-3.73	Down	--	virulence protein
gene_0977	0.02	-5.32	Down	*sptP*	Type III secretion injected virulence protein
gene_0968	0.01	-6.57	Down	--	oxygen-regulated invasion protein OrgB
gene_0969	0.03	-5.22	Down	--	oxygen-regulated invasion protein OrgA
gene_3867	0.06	-3.97	Down	--	adhesin/invasin protein PagN
gene_0976	0.01	-7.15	Down	--	Invasion protein IagB precursor
gene_1045	0.05	-4.33	Down	*sopD*	SPI-1 type III secretion system effector SopD
gene_3924	0.02	-5.81	Down	*sopD2*	SPI-2 type III secretion system effector SopD2
gene_4211	0.27	-1.87	Down	*fliR*	flagellar type III secretion system protein FliR
gene_4212	0.29	-1.78	Down	*fliQ*	flagellar biosynthetic protein FliQ
gene_4213	0.17	-2.58	Down	*fliP*	flagellar biosynthetic protein FliP
gene_4214	0.1	-3.38	Down	*fliO*	flagellar protein FliO
gene_4215	0.12	-3.07	Down	*fliN*	flagellar motor switch protein FliN
gene_4216	0.14	-2.86	Down	*fliM*	flagellar motor switch protein FliM
gene_4217	0.18	-2.48	Down	*fliL*	flagellar basal body-associated protein FliL
gene_4218	0.17	-2.54	Down	*fliK*	flagellar hook length control protein FliK
gene_4219	0.16	-2.66	Down	*fliJ*	Structure of FliJ, a soluble component of flagellar type III export apparatus

Gene_ID	*ompR*_VS_*wt*_Fold Change	*ompR*_VS_*wt*_log2Fold Change	Regulate	Gene_Type	NR_Annotation
gene_4220	0.25	-2.03	Down	*fliI*	flagellum-specific ATP synthase
gene_4221	0.22	-2.19	Down	*fliH*	flagellar assembly protein FliH
gene_4222	0.23	-2.13	Down	*fliG*	flagellar motor switch protein FliG
gene_4223	0.28	-1.83	Down	*fliF*	flagellar M-ring protein FliF
gene_4224	0.16	-2.62	Down	*fliE*	flagellar hook-basal body complex protein FliE
gene_4233	0.25	-2.01	Down	*fliB*	lysine-N-methylase
gene_0367	0.14	-2.83	Down	*nmpC*	porin OmpD
gene_0412	0.2	-2.31	Down	--	porin OmpC
gene_0467	0.33	-1.59	Down	*rstA*	two-component system response regulator RstA
gene_0546	0.06	-4.09	Down	*ssrB*	two component system response regulator
gene_0551	0.13	-2.90	Down	*ttrS*	two-component system sensor histidine kinase TtrS
gene_0650	0.47	-1.10	Down	*mipA*	MipA/OmpV family protein
gene_0700	0.19	-2.37	Down	*phoQ*	two-component system sensor histidine kinase PhoQ
gene_1733	0.48	-1.04	Down	--	OmpA family lipoprotein
gene_1876	0.36	-1.49	Down	*envZ*	two-component system sensor histidine kinase EnvZ
gene_0007	0.27	-1.89	Down	*sdiA*	transcriptional regulator SdiA
gene_0917	0.37	-1.45	Down	*emrB*	Inner membrane component of tripartite multidrug resistance system
gene_0916	0.45	-1.16	Down	*emrA*	multidrug resistance protein A
gene_0669	0.05	-4.43	Down	--	LuxR family transcriptional regulator
gene_1604	0.21	-2.22	Down	*nepI*	purine ribonucleoside efflux pump NepI

Gene_ID	*ompR*_VS _*wt*_Fold Change	*ompR*_VS_ *wt*_log2Fold Change	Regulate	Gene_Type	NR_Annotation
gene_1791	0.32	-1.63	Down	*ABC-2.TX*	HlyD family efflux transporter periplasmic adaptor subunit
gene_2171	0.35	-1.52	Down	*acrB*	efflux RND transporter permease subunit
gene_2713	0.49	-1.03	Down	*kefC*	glutathione-regulated potassium-efflux system protein KefC
gene_4246	0.49	-1.03	Down	*kefG*	glutathione-regulated potassium-efflux system ancillary protein KefG
gene_4403	0.3	-1.75	Down	*rpoE*	RNA polymerase sigma factor RpoE
gene_0975	0	-8.36	Down	*hilA*	transcriptional regulator HilA
gene_0397	0.03	-5.29	Down	--	PhoPQ-regulated protein
gene_0699	0.14	-2.82	Down	*phoP*	DNA-binding response regulator
gene_1543	0.49	-1.04	Down	--	PhoP regulatory network protein YrbL
gene_0700	0.19	-2.37	Down	*phoQ*	two-component system sensor histidine kinase PhoQ
gene_0499	0.21	-2.25	Down	*SOD1*	superoxide dismutase
gene_0617	0.09	-3.41	Down	*katE*	catalase HPII
gene_3409	0.47	-1.10	Down	*proP*	L-Proline transporter ProP
